# Comparison of Gold Biosensor Combined with Light Microscope Imaging System with ELISA for Detecting *Salmonella* in Chicken after Exposure to Simulated Chilling Condition

**DOI:** 10.4014/jmb.2212.12011

**Published:** 2023-01-12

**Authors:** Mi-Kyung Park

**Affiliations:** 1 School of Food Science and Biotechnology, Kyungpook National University, Daegu 41566, Republic of Korea; 2 Food and Bio-Industry Institute, Kyungpook National University, Daegu 41566, Republic of Korea

**Keywords:** *Salmonella*, polyclonal antibody, enzyme-linked immunosorbent assay, gold biosensor, light microscope imaging, chicken

## Abstract

In this study, the performance of a gold biosensor combined with light microscope imaging system (GB-LMIS) was comparatively evaluated against enzyme-linked immunosorbent assay (ELISA) for detecting *Salmonella* under simulated chilling condition. The optimum concentration of anti-*Salmonella* polyclonal antibodies (pAbs) was determined to be 12.5 and 100 μg/ml for ELISA and GB-LMIS, respectively. GB-LMIS exhibited a sufficient and competitive specificity toward three tested *Salmonella* among only. To mimic a real-world situation, chicken was inoculated with *Salmonella* cocktail and stored under chilling condition for 48 h. The overall growth of *Salmonella* under chilling condition was significantly lower than that under non-exposure to the chilling condition (*p* < 0.05). No significant differences in bacterial growth were observed between brain heart infusion and brilliant green broth during the enrichment period (*p* > 0.05). Finally, both GB-LMIS and ELISA were employed to detect *Salmonella* at every 2-h interval. GB-LMIS detected *Salmonella* with a competitive specificity by the direct observation of bacteria on the sensor using a charge-coupled device camera within a detection time of ~2.5 h. GB-LMIS is a feasible, novel, and rapid method for detecting *Salmonella* in poultry facilities.

## Introduction

Poultry is the second highest source of meat consumption, and its demand is continuously increasing due to rapid production under automated processing facilities and affordable price [1]. Moreover, poultry is the most common source of *Salmonella*, which is a threat to human health worldwide [2-5]. A study reported that 279 of 1,114 outbreaks (25%) from 1998 to 2012 in the U.S. were linked to poultry [1, 6]. Of these 279 outbreaks, 149 could be traced back to several confirmed pathogens, including *Salmonella* spp. (43%), *Clostridium perfringens* (26%), *Campylobacter* (7%), *Staphylococcus aureus* (5%), *Bacillus cereus* (3%), and *Listeria monocytogenes* (3%). Several preventive and hygiene measures have been developed and implemented for controlling *Salmonella* in processing facilities using post-chilling immersion tank as well as spraying applications with various antimicrobials, such as sulfuric acid, sodium sulfate, sodium chloride, calcium hypochlorite, organic acids, and bacteriophage solution [1]. However, these measures are limited by low effectiveness and the rapid resistance development of *Salmonella* (~1 log reduction) [7, 8].

Although a conventional method has been recognized as the “gold standard of detection” [9], its time-consuming and labor-intensive procedures remain problematic for employing it on-site [5, 10, 11]. Numerous biosensor methods have been developed for use in clinical diagnostics, environmental monitoring, and foodborne pathogen detection [12-15]. A biosensor consists of a bioreceptor for identifying and binding with a specific target and a transducer for integrating the binding of the bioreceptor with the target on the sensor platform [15-17]. In the past two decades, antibodies, as one of the major bioreceptors, have been commonly used in various biosensor methods due to their excellent binding capability with each target pathogen [18]. However, only a few biosensors have been practically used in food processing facilities for monitoring and detecting foodborne pathogens [19]. Non-specific bindings of the food matrix, when employed in a food sample, could interfere or block the binding of target pathogens with bioreceptors, resulting in a significant reduction in sensitivity, specificity, and reliability of biosensor methods [20-22].

Herein, a gold biosensor combined with light microscope imaging system (GB-LMIS) was developed by our research group [12, 23]. The method employing the GB-LMIS is based on the binding of antibodies with target pathogens on a gold sensor, following almost the same principle as that of the enzyme-linked immunosorbent assay (ELISA). The difference is the introduction of a gold sensor in GB-LMIS for the immobilization of antibodies. Moreover, no extra enzymes or secondary antibodies are required for quantifying target pathogens. In the GB-LMIS, a square-cut section of glass is coated with a nanometer-scale, thin gold layer for facilitating antibody binding. Upon placing the antibody-immobilized sensor in food, the antibodies on the sensor bind with a foodborne pathogen. The bound target pathogen on the sensor is visualized and enumerated using a light microscope equipped with a charged-coupled device (CCD) camera. So far, the GB-LMIS has been employed to detect *Escherichia coli* O157:H7 in turnip greens [23] and *L. monocytogenes* in chicken [12] as target pathogens. Using the binding of a foodborne pathogen with a specific antibody on the sensor, the GB-LMIS can capture and visualize these target pathogens, thereby aiding in differentiation of target pathogens from several unavoidable food matrices. This study aimed to compare the performance of the GB-LMIS and ELISA for detecting *Salmonella* in chicken using anti-*Salmonella* polyclonal antibodies (pAbs) as an on-site applicable detection method in poultry processing facilities.

## Materials and Methods

### Bacteria and Culture Condition

The bacterial species tested in this study (Table 1) were obtained from the Food Microbiology Laboratory at Auburn University (USA). *Salmonella* Typhimurium and *S*. Enteritidis were incubated in 20 ml of Trypticase Soy Broth (TSB, Difco Laboratories Inc., USA) for 16 h at 37°C. After cultivation, each bacterial culture was washed 3 times with phosphate-buffered saline (PBS, pH 7.2, Sigma-Aldrich Co., USA) by centrifugation at 5,000 ×*g* for 5 min. The precipitated cells were resuspended in PBS and each bacterial concentration was determined using a preconstructed standard curve. A *Salmonella* cocktail was prepared by mixing equal amounts of *S*. Typhimurium and *S*. Enteritidis. Other bacterial species (Table 1), except *Listeria* spp., were cultured in TSB whereas two strains of *Listeria* were cultured in TSB containing 0.6% yeast extract (TSBYE) for 16 h at 37ºC. After cultivation, each bacterial culture was washed and centrifuged for preparing a bacterial suspension according to the abovementioned procedures.

### Purification of Anti-*Salmonella* pAbs

Ascites fluid with anti-*Salmonella* pAbs (Hybridomas Laboratory, Auburn University) was produced from a white rabbit (New Zealand) against *Salmonella* cocktail and purified through ammonium sulfate precipitation and protein A affinity column chromatography (Sigma Chemical Co.). After confirming its purity using 12%sodium dodecyl sulfate-polyacrylamide gel electrophoresis (SDS–PAGE), the concentration of purified anti-*Salmonella* pAbs was finally determined using the Bradford method [12].

### Preparation of Gold Sensor

A glass square (5 mm × 5 mm) with a thickness of 0.17 mm was cut using a micro-dicing saw (MPE Inc., USA). After ultrasonic cleaning, the sensor was cleaned further using acetone, ethanol, and filtered distilled water (FDW). The cleaned sensor was coated with Cr and Au with a thickness of 40 nm using a Pelco SC-6 sputter (Ted Pella Inc., USA).

### Reactivity and Specificity of Anti-*Salmonella* pAbs Using ELISA

For the reactivity of anti-*Salmonella* pAbs, 100 μl of *Salmonella* cocktail (10^8^ CFU/ml) was placed in an ELISA plate (Costar, USA) and incubated at 37°C for 1 h. After washing 3 times with 200 μl of PBS containing 0.1% Tween 20 (PBST), the unbound area of the wells was blocked with 200 μl of 1% bovine serum albumin (BSA, Sigma-Aldrich Co.) for 1 h at 37°C, followed by washing thrice with PBST. An aliquot of 100 μl of anti-*Salmonella* pAbs (0.6–400 μg/ml) was stored at room temperature (RT) for 2 h. After washing with PBST, 100 μl of alkaline phosphatase-conjugated anti-rabbit goat IgG (0.5 μg/ml, Sigma-Aldrich Co.) was added and incubated for 1 h at RT. Finally, after washing 3 times with PBST, 100 μl of p-nitrophenyl phosphate (p-npp, Sigma Chemical Co.) was added as a substrate and the absorbance of each well was measured at 405 nm using a microplate reader (Thermo Labsystems, Finland). After 15 min of incubation in the dark at RT, the absorbance was measured again. The absorbance results were expressed as the means of the absorbance difference with standard deviations. For determining the specificity of anti-*Salmonella* pAbs, 100 μl of each bacterial suspension was incubated at 37°C for 1 h. After washing with PBST, the unbound area was blocked with 200 μl of 1% BSA at 37°C for 1 h. Finally, 100 μl of anti-*Salmonella* pAbs (50 μg/ml) was added and the abovementioned procedures were performed. A cutoff value was determined based on the mean of the negative control plus 0.25 OD units [24].

### Reactivity and Specificity of Anti-*Salmonella* pAbs Using GB-LMIS

A gold sensor was immobilized with various concentrations of 100 μl of anti-*Salmonella* pAbs (3.0–400 μg/ml) against the *Salmonella* cocktail to evaluate their reactivity. The gold sensor was immobilized with the same amount of anti-*Salmonella* pAbs (100 μg/ml) against various foodborne pathogens for determining their specificity. A control sensor was also immobilized with 100 μl of DW. After incubation at 37°C for 1 h, the sensor was washed 3 times with PBS and the unbound areas of the sensor were blocked with 100 μl of 1% BSA at RT for 1 h. Then, the blocked sensor was washed 3 times with PBS and air-dried for use as an immunosensor. The immunosensor was then incubated with 100 μl of *Salmonella* cocktail (10^8^ CFU/ml) for determining the reactivity of anti-*Salmonella* pAbs with other bacterial suspensions (10^8^ CFU/ml) as well as their specificity at 22°C for 1 h. After incubation, the immunosensor was washed with FDW, dried, and then treated with 4% OsO_4_ (Sigma-Aldrich Co.) for 1 h. The bacteria captured on the immunosensor were observed under a light microscope equipped with a CCD camera (Nikon Eclipse L 150, Nikon Instruments Inc., USA) at 1,000× magnification. The captured bacterial images were enumerated from 10 selected areas on the surface of the immunosensor. The detected number of bacteria on the immunosensor was determined from the average number of bacteria counted in each area and expressed as cell per mm^2^ (cell/mm^2^).

### Comparison of GB-LMIS with ELISA for *Salmonella* Detection in Chicken After Exposure to Chilling Conditions

Chicken skins were randomly collected from Koch Food Company (USA) and sliced into 10 cm × 10 cm samples. To minimize contamination, chicken skin was washed with 200 ppm chlorine solution (Sigma-Aldrich Co.) and sterilized DW. Then, 200 ml of the *Salmonella* cocktail was inoculated onto the chicken at concentrations ranging from 10^1^ to 10^3^ CFU/100 cm^2^. An equal amount of PBS was added onto other chicken skins as negative controls. The inoculated chicken skins were dried under a biosafety cabinet for bacterial attachment and placed in an Erlenmeyer flask prior to further incubation in a refrigerator (4°C) for 48 h. Next, 100 ml of brain heart infusion (BHI, EMD Science, Germany) or brilliant green (BG, Difco Laboratories Inc.) broth was added to each flask and incubated at 37°C in an orbital shaker at 250 rpm. Then, 100 μl of sample was collected from BHI and BG broths at 0, 2, 4, and 6 h, and the resuscitated bacterial population was measured using xylose lysine deoxycholate agar (Difco Laboratories Inc.) and recorded as log CFU/chicken for comparison. Subsequently, 20 ml of samples were obtained from both broths and washed 3 times by centrifugation at 4,000 ×*g* for 20 min. After resuspending with 1 ml of PBS, 100 μl of *Salmonella* suspension was used for ELISA and GB-LMIS, as described in the previous section. The results are expressed as log CFU/chicken for the comparison.

### Statistical Analysis

Experimental results are expressed as mean ± SD. Comparisons between various treatments and/or groups were performed using one-way analysis of variance with Tukey's multiple comparison test and Student’s paired *t*-test. Statistical analysis was performed using GraphPad InStat v.3 (GraphPad, USA).

## Results and Discussion

**Fig. 1:**
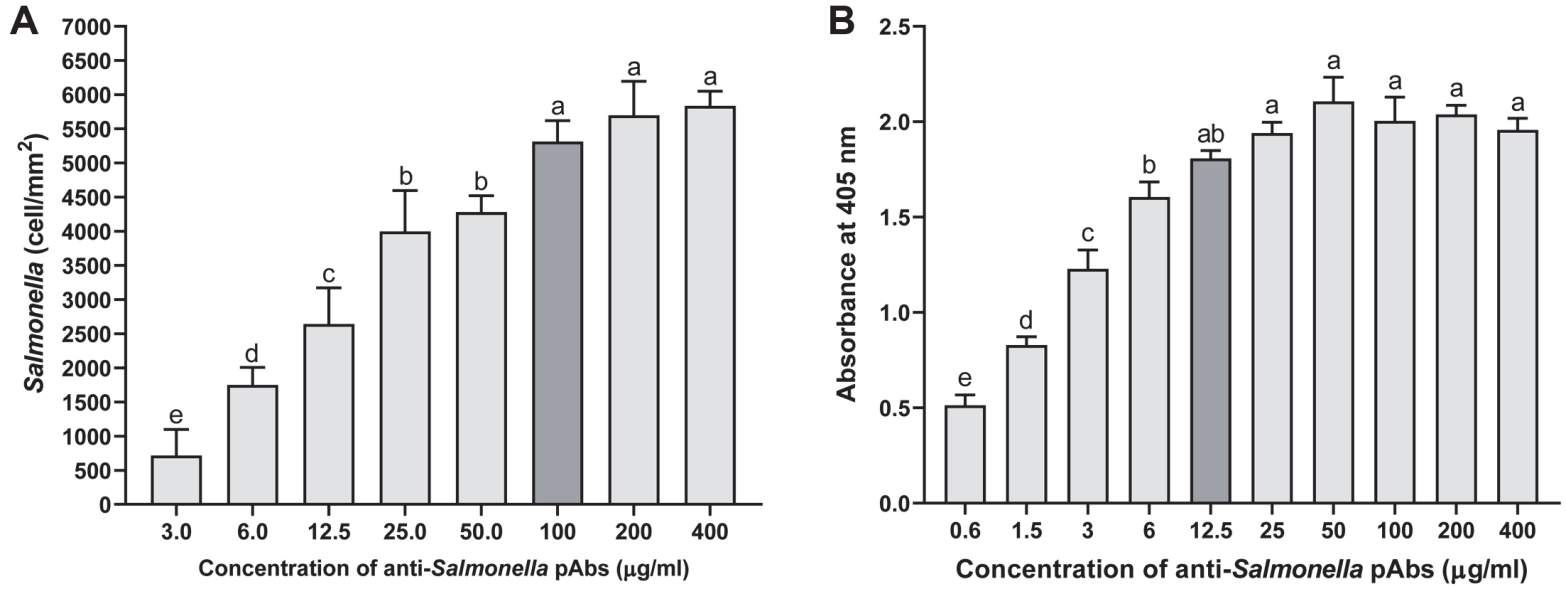
Reactivity of anti-*Salmonella* polyclonal antibody measured using (A) GB-LMIS (*n* = 30) and (B) ELISA (*n* = 3). The letters (a–e) indicate statistically significant differences compared with other treatments (*p* < 0.05).

The successful performance of an antibody-based detection method is absolutely dependent on the reactivity and specificity of the antibody. Anti-*Salmonella* pAbs (6.5 mg/ml) were purified through ammonium sulfate precipitation and protein A affinity column chromatography. The reactivity of anti-*Salmonella* pAbs was determined using GB-LMIS and ELISA (Fig. 1). The binding of *Salmonella* on the immunosensor significantly increased up to antibody concentrations of 100 μg/ml (*p* < 0.05) and then remained steady, indicating no significant differences. Therefore, the optimum concentration of anti-*Salmonella* pAbs was 100 μg/ml for GB-LMIS. Similar to the result of ELISA, the reactivity of anti-*Salmonella* pAbs with *Salmonella* was significantly increased up to 12.5 μg/ml (Fig. 1B) (*p* < 0.05). Further, the increase in antibody concentrations did not exhibit a significant and positive influence on the binding reactivity with *Salmonella*. Therefore, the optimum concentrations of anti-*Salmonella* pAbs were determined to be 12.5 and 100 μg/ml for ELISA and GB-LMIS, respectively. The optimum concentration of anti-*Salmonella* pAbs (100 μg/ml) for GB-LMIS was approximately 8-fold higher than that for ELISA.

Since poultry may coexist with other microorganisms, such as *Micrococci*, *Pseudomonas*, *E. coli*, *L. monocytogenes*, *S. aureus*, *Campylobacter jejuni*, and *Salmonella*, the antibody needs to react with a target among other heterogeneous microorganisms [25, 26]. For comparing the specificity of anti-*Salmonella* pAbs (Table 1), the cutoff value of ELISA was 0.379 based on the mean of the negative control (0.129) plus 0.25 OD units [24]. Anti-*Salmonella* pAbs demonstrated a significantly greater specificity against all *Salmonella* strains tested using both methods (*p* < 0.001). Although few bacteria were captured on the immunosensor, they were negligible and similar results were obtained from the control sensors (devoid of anti-*Salmonella* pAbs). Thus, anti-*Salmonella* pAbs exhibited a sufficient specificity against *Salmonella* only by providing a greater absorbance and bacterial bindings on the immunosensor for ELISA and GB-LMIS. Purified anti-*Salmonella* pAbs demonstrated a sufficient specificity against *S*. Typhimurium, *S*. Enteritidis, and *S*. Heidelberg, which are representative strains in poultry [27, 28]. More importantly, GB-LMIS exhibited a competitive and robust specificity compared with ELISA.

Following the US regulations, poultry carcasses should be chilled to ≤ 4.4°C for a certain period to ensure a high-quality and safe product [29]. Under similar chilling condition, chicken was inoculated with *Salmonella* cocktail prior to placing at 4°C for 48 h. A previous study [30] revealed that the minimum growth temperature of *Salmonella* in poultry was 5°C. Thus, it was hypothesized that *Salmonella* inoculated on chicken after exposure to 4°C for 48 h might be injured. An enrichment procedure is inevitably required to reach the detectable number of bacteria (detection limit) and resuscitate injured *Salmonella* to prevent false-negative results. The enrichment procedure will increase the number of *Salmonella* and recover the injured cells during the chilling period, although the enrichment period may increase the total detection time and diminish the on-site applicability of GB-LMIS. As our previous study [30] showed that BHI and BG broths were the most efficient non-selective and selective media for *Salmonella* on chicken, respectively, these two media were selected for culturing *Salmonella*.

**Fig. 2:**
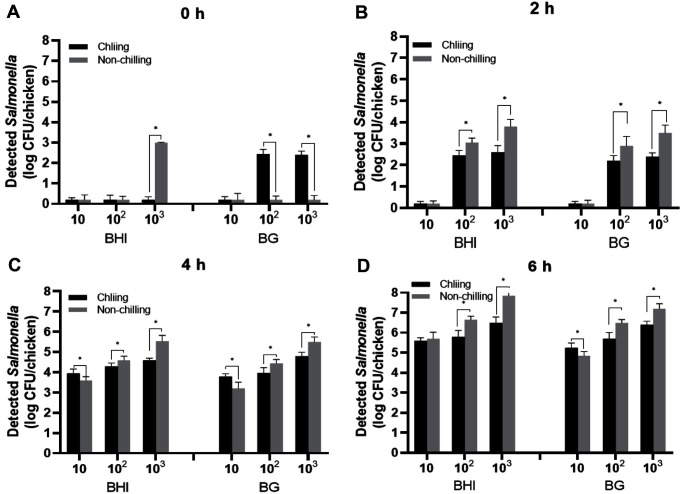
Growth and recovery of *Salmonella* inoculated on chicken using BHI and BG broth. (*) indicates statistically significant differences between chilling and non-chilling treatments (*p* < 0.05).

The populations of *Salmonella* in BHI and BG enrichment broths after chilling at 4°C for 48 h were compared with those that were not exposed to chilling condition at every 2-h interval (Fig. 2). As the enrichment time and inoculation concentration increased, the bacterial growth increased. However, the overall growth of *Salmonella* under chilling condition was significantly lower than that under non-exposure to chilling condition (*p* < 0.05). No significant differences in bacterial growths were observed between BHI and BG broth during the whole incubation time, as long as the initially inoculated bacterial concentration was the same (*p* > 0.05). These results confirmed the suitability of both enrichment broths for the recovery of *Salmonella* injured through chilling and provided an approximate estimation of bacterial growth rate during the 6-h enrichment period.

**Fig. 3:**
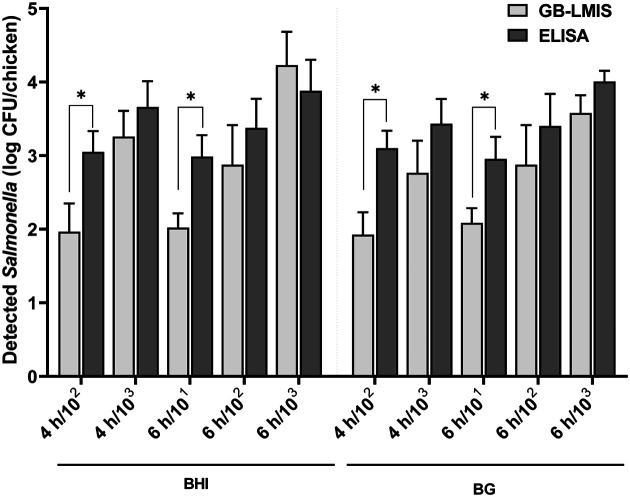
The number of *Salmonella* detected from the inoculated chicken after enriching in BHI and BG broth using GB-LMIS and ELISA. The number of *Salmonella* detected using ELISA was obtained by placing the OD result into the equation (Y = 0.159 × − 0.189). (*) indicates that there was a significant difference between BHI and BG samples within the same inoculum and enrichment time at *p* < 0.05. Vertical bars represent the standard deviation (*n* = 45).

Finally, both methods were employed to detect *Salmonella* at a 2 h interval (Fig. 3). Unlike the GB-LMIS, ELISA requires conversion work to represent the number of *Salmonella* from OD values using an equation (Y = 0.159 × - 0.189) (Table 2). Based on a previous study [23], the detection limit of the GB-LMIS for *Salmonella* detection was determined to be 10^3^ CFU/sensor. Both methods could detect *Salmonella* on chicken samples with initial inoculation concentration of 10^2^ and 10^3^ CFU on chicken and a 4-h enrichment period, and those with initial concentration of 10^1^, 10^2^, and 10^3^ CFU and a 6-h enrichment period. Following the pattern of slightly greater populations of *Salmonella* in BHI (Fig. 2), the detected bacterial numbers in BHI were greater than those in BG for both methods. Although a greater number of *Salmonella* was detected using ELISA than GB-LMIS, no significant differences were observed between the tested methods, except for a chicken sample with initial inoculation concentration of 10^2^ CFU and a 4-h enrichment period, and those with initial inoculation concentration of 101 CFU and a 6-h enrichment period (*p* < 0.05). Higher numbers of *Salmonella* were detected using ELISA because the quantification of bacteria relies on a sensitive enzyme reaction. The enzyme used in ELISA is generally conjugated to secondary antibodies, thereby requiring a substrate for reacting with the enzyme. The introduction of secondary antibody and substrate in ELISA requires additional incubation time and washing procedure. Meanwhile, the GB-LMIS could detect and visualize *Salmonella* without additional chemicals, thereby demonstrating its competitive and comparable detection capability in an easy and simple manner.

There was some potential influence of media (broth) and/or interference of the food matrix on the performance of both methods. Other studies [31-33] showed that Rappaport–Vassiliadis [27] medium reduced the sensitivity of ELISA, although the RV medium was more effective in increasing the number of *Salmonella* compared with other media. In a previous study [31], an unknown component of RV medium was found to impact the expression of the antigenic epitope, thereby interfering with the binding of antigen and antibody. The GB-LMIS captured the target pathogen based on the antigen and antibody binding on the sensor and enabled visualization in a user-friendly and rapid manner. As shown in Table 3, the GB-LMIS exhibited a competitive and robust specificity for detecting *Salmonella* without any aid of enzyme labeling to the antibody for enhancing the reactivity as compared with ELISA. GB-LMIS is cost effective because there is no need for enzyme or fluorescent conjugation to quantify the bacterial bindings. In contrast, the necessity of a label-conjugated secondary antibody in ELISA increased the detection time and decreased its practicability as an on-site applicable method in the food industry [14, 23]. Although the required concentration of antibodies for the GS-LMIS was 8-fold higher than that required for ELISA, the GB-LMIS overcame the limitation of ELISA without unnecessary conversion procedure after the measurement of OD. Thus, the GB-LMIS was a more cost-effective and time-effective method as it decreased the detection cost and time from ~ $1.80 to ~ $0.31 and ~5.5 to ~2.5 h, respectively, excluding the enrichment period. Although the enrichment period increased the overall detection time, the bacterial concentration should reach at least a detectable level, regardless of the detection method. Hence, it can be concluded that the GB-LMIS is a feasible, novel, and rapid method for detecting *Salmonella* in poultry facilities.

## Figures and Tables

**Table 1:** Specificity of purified anti-*Salmonella* polyclonal antibodies using GB-LMIS and ELISA.

Bacteria	Detection method	

	GB-LMIS (cell/mm^2^)	ELISA
*Salmonella* Typhimurium ATCC 13311	23,127 ± 3,264^a^	1.693 ± 0.054^a^
*S.* Enteritidis	28,221 ± 2,997^a^	1.724 ± 0.028^a^
*S.* Heidelberg	20,765 ± 4,375^a^	1.166 ± 0.19^a^
*Citrobacter freundii* ATCC 8090	68 ± 82^b^	0.154 ± 0.014^b^
*Escherichia coli* O157:H7	325± 205^b^	0.218 ± 0.017^b^
*E. coli* ATCC 700599	371 ± 237^b^	0.258 ± 0.027^b^
*Klebsiella oxytoca* ATCC13182	217 ± 178^b^	0.224 ± 0.035^b^
*Listeria monocytogenes* H7738	151 ± 169^b^	0.238 ± 0.009^b^
*L. innocua* ATCC33090	251± 257^b^	0.119 ± 0.012^b^
*Micrococcus luteus* ATCC 10240	169 ± 127^b^	0.292 ± 0.013^b^
*Pseudomonas aeruginosa* ATCC 10145	95 ± 152^b^	0.164 ± 0.013^b^
*Staphylococcus aureus* ATCC 6538	75 ± 89^b^	0.254 ± 0.037^b^
*Shigella sonnei* ATCC 25931	69 ± 73^b^	0.226 ± 0.012^b^
*Yersinia enterocolitica* ATCC 23715	97 ± 99^b^	0.194 ± 0.022^b^
*Vibrio parahaemolyticus* ATCC 17802	89 ± 73^b^	0.214 ± 0.011^b^
Negative control^1)^	19 ± 66^b^	0.129 ± 0.013^b^

^1)^Indicates the exposure of gold sensor (devoid of immobilization of anti-*Salmonella* pAbs) to *Salmonella* cocktail.

The letters (a and b) indicate statistically significant differences within a column (*p* < 0.001).

**Table 2:** Detection of *Salmonella* in chicken after exposure to chilling condition using ELISA.

Incubation time	BHI			BG		

	10^1^	10^2^	10^3^	10^1^	10^2^	10^3^
2 h	0.170 ± 0.000^*^	0.182 ± 0.040	0.190 ± 0.032	0.188 ± 0.010	0.203 ± 0.020	0.216 ± 0.039
4 h	0.236 ± 0.041	0.296 ± 0.043	0.393 ± 0.026	0.229 ± 0.043	0.304 ± 0.041	0.357 ± 0.044
6 h	0.286 ± 0.040	0.348 ± 0.034^**^	0.428 ± 0.047	0.281 ± 0.034	0.352 ± 0.060	0.448 ± 0.044

(*) indicates absorbance differences measured before and after 15-min incubation (*n* = 3).

**Table 3:** Performance comparison of GB-LMIS and ELISA.

	GB-LMIS	ELISA
Specificity	Target specific	Target specific
Reactivity	Less sensitive	More sensitive
Antibody dose	100 μg/ml	12.5 μg/ml
Bacterial quantification	Direct quantification	Necessity of extra converting
Detection time	~2.5 h	~5.5 h
Media effect	Robust	Susceptible
